# Fast Curing Bio-Based Phenolic Resins via Lignin Demethylated under Mild Reaction Condition

**DOI:** 10.3390/polym9090428

**Published:** 2017-09-07

**Authors:** Jiongjiong Li, Jizhi Zhang, Shifeng Zhang, Qiang Gao, Jianzhang Li, Wei Zhang

**Affiliations:** 1 Ministry of Education (MOE) Key Laboratory of Wooden Material Science and Application, Beijing Key Laboratory of Wood Science and Engineering, School of Materials Science and Technology, Beijing Forestry University, Beijing 100083, China; luckyjiong@126.com (J.L.); shifeng.zhang@bjfu.edu.cn (S.Z.); gaoqiang@bjfu.edu.cn (Q.G.); 2 Key Laboratory for Liquid-Solid Structural Evolution and Processing of Materials (MOE), School of Materials Science and Engineering, Shandong University, Jinan 250061, China; zjzvip@sdu.edu.cn

**Keywords:** alkali lignin, demethylation efficiency, demethylation mechanism, bio-based phenolic resins

## Abstract

Demethylation technique has been used to enhance lignin reactivity for preparation of phenolic resins. However, the demethylation efficiency and the demethylated lignin (DL) reactivity were still unsatisfactory. To improve the demethylation efficiency, alkali lignin was demethylated under different mild conditions using sodium sulfite as a catalyst. Lignin and DL were characterized by ^1^H-NMR (nuclear magnetic resonance) and Fourier transform infrared (FT-IR) spectroscopy to determine the demethylation mechanism. With the demethylation of lignin, the methoxyl group content decreased from 1.93 m mol/g to 1.09 m mol/g, and the phenolic hydroxyl group content increased from 0.56 m mol/g to 0.82 m mol/g. These results revealed that methoxyl groups were attacked by SO_3_^2−^, and some methoxyl groups were converted to phenolic hydroxyl groups by a nucleophilic substitution reaction, generating DL with high reactivity. The chemical properties of lignin-based phenolic resins were studied by ^13^C-NMR and FT-IR spectroscopy, and their physical properties were also investigated. The results indicated that lignin-based phenolic resins exhibited faster curing rate and shorter gel time. In addition, the bonding strength increased from 0.92 MPa to 1.07 MPa, and the formaldehyde emission decreased from 0.58 mg/L to 0.22 mg/L after lignin demethylated at the optimum condition.

## 1. Introduction

In nature, lignin is the second most abundant renewable raw material available after cellulose. It is an amorphous three-dimensional phenyl-propanol biopolymer containing three different types of phenyl-propane units (H, G, and S), crosslinked by C–C bonds (β–1, β–5, β–β, and 5–5) and C–O–C bonds (4–O–5, α–O–4, and β–O–4) [1,2,3,4]._ENREF_1_ENREF_4 Despite high availability of lignin, few lignin-based commodity materials are available due to its complex structure, broad chemical differences, and low reactivity. At present, lignin has been studied for two general uses; besides, most is used for process heat or for co-firing in power plants. The first approach to the general use of lignin is production of valuable small molecular weight aromatic compounds by depolymerization or refinery process [5,6,7,8]._ENREF_5 _ENREF_7The second is its use as a modifier and/or an ingredient in the synthesis of commercial polymer products (e.g., adhesives, resins, and plastics) [9,10]. Nevertheless, besides the robust and amorphous chemical structure of lignin, it possesses diverse properties depending on temperature, humidity and ultraviolet (UV) exposure [11]. These properties make lignin unsuitable starting material for the preparation of current commercial polymer products. Therefore, it is still necessary to prepare lignin with some degree of controlled modification. 

In recent decades, many lignin modification methods such as demethylation [12,13,14], methylolation [15,16], and phenolation [17,18] have been explored to increase the reactivity of lignin. Among these modification strategies, lignin demethylation is the most efficient and promising method by converting methoxyl groups into phenolic hydroxyl groups, generating catechol moieties in lignin framework [19]. Hydrogen iodide [20], hydrogen chloride [11], and other diverse hydrolysis reactions in acidic conditions [11] have been employed to remove methoxy groups and cleave ether groups through demethylation route. However, many of these results were inconclusive because of the restricted analytical methods. Additionally, there were few studies focused on the demethylation of methoxyl groups using fungal (white rot fungi or brown rot fungi) [21] and bacteria [22] (*Pseudomonas* or *Sphingomonas*); however, these processes will inevitably depolymerize lignin into small molecules and the ability of laccase to degrade lignin without mediators has been in doubt [12,23].

In recent years, the demethylation of methoxy groups using sulfur dioxide-mediated methods has also been reported [19,24]. Wu et al. [24] demonstrated that a 40% reduction in methoxyl group content and a 85% increase in phenolic hydroxyl group content could be achieved after lignin demethylation using sulfur dioxide as a demethylation catalysts. Although these methods are capable of demethylating lignin, the major disadvantages of these methods are the use of high temperatures (up to 225 °C), long processing time (up to 6 h), and high pressures. Alternatives to these approaches should increase lignin reactivity while avoiding the use of such extreme reaction conditions. 

Sulfite process is one of the main type of chemical pulping in pulp and paper industries [25]. During the alkaline sulfite pulping, hydroxyl and sulfite ions can engage in nucleophile substitution reaction with lignin, resulting in the formation of sulfite pulp. In addition, sulfite ion can cleave the aryl methyl ethers of aromatic compounds by nucleophilic substitution reaction, generating a remarkable reduction in methoxyl groups and an enhanced hydrophilicity of lignin. Hence, the reactivity of crop-based lignin was improved by decreasing its methoxyl group content. Although alkaline sulfite exhibits a positive effect on the demethylation of lignin, few studies using lignin directly as a substrate have been conducted to investigate its reactivity. Lignin was demethylated under atmospheric pressure and low temperature using four different demethylation catalysts observed in previous experiment of Li et al. [12]. It was found that the methoxyl groups of lignin were transformed by the addition of demethylation catalysts into phenolic hydroxyl groups, and the demethylated lignin possessed more phenolic hydroxyl groups on the lignin molecule and higher molecular reactivity. In addition, the demethylated lignin was suitable for the preparation of lignin-based phenolic resins. Nevertheless, some of the demethylation catalysts such as powdered sulfur and n-dodecyl mercaptant have low aqueous solubility, which would restrict the demethylation process due to its poor dispersibility. The other demethylation catalyst, sodium hydrosulfide, possesses pungent odor, which makes it unsuitable for industrial application. Sodium sulfite seems to be a suitable catalyst for the demethylation of lignin with higher demethylation ability than other catalysts. However, the demethylation efficiency and the reactivity of demethylated lignin (DL) still unsatisfactory. In addition, the demethylation reaction mechanisms have not been extensively explored. 

In this work, we sought to enhance the demethylation performance by demethylating lignin using sodium sulfite as a catalyst under mild reaction conditions. The obtained lignin was characterized by Fourier transform infrared (FT-IR) spectroscopy and ^1^H-NMR (nuclear magnetic resonance), and the most suitable lignin was used for the preparation of fast curing bio-based phenolic resins. The aim of this study was to improve the demethylation efficiency and further to establish the demethylation reaction mechanism for lignin demethylation. The evaluation of the demethylation parameters was based on the quantification of functional groups that are relevant for the DL products and the properties of lignin-based phenolic resins. 

## 2. Materials and Methods 

### 2.1. Materials

Formaldehyde (aqueous solution, 37 wt %), solid phenol, and sodium hydroxide were AR grade reagents obtained from Beijing Chemical Reagents Co., Ltd. (Beijing, China). Solid urea was an industrial-grade reagent purchased from Lanyi Chemical Co., Ltd. (Nanjing, China). Sodium sulfite (Na_2_SO_3_, 97 wt %) were provided by Guangdong Xilong Chemical Factory (Shantou, China). Alkali lignin were obtained from Xinyi Feihuang Chemical Co., Ltd. (Xuzhou, China).

In this work, alkali lignin was used for the preparation of fast curing bio-based phenolic resins. First, alkali lignin was purified with dilute acid to remove carbohydrates and ashes; second, the purified alkali lignin was demethylated; third, the purified alkali lignin and the demethylated alkali lignin were used for the preparation of phenolic resins. All the specimens analyzed in this work are listed as below.

Lignin stands for the purified alkali lignin; DL stands for the demethylated alkali lignin; PF stands for the phenol-formaldehyde resins; LPF stands for the phenolic resins modified with the purified alkali lignin; DLPF stands for phenolic resins modified with the demethylated alkali lignin.

### 2.2. Purification of Lignin

Alkali lignin was purified prior to the demethylation reaction. In a typical purification process, 10 g of lignin was dissolved in 100 g of distilled water, and then the mixture was acidified with sulfuric acid (10 wt %) until reaching a pH around 2. A solid precipitated that was isolated by centrifugation using a laboratory centrifuges at 4000 rpm for 6 min. After that, the isolated residual was washed with distilled water until the pH was adjusted to neutral. Finally, the purified lignin was freeze dried by placing them in a vacuum freeze-drier (Sihuan LGJ-25C, Sihuan Co., Ltd., Beijing, China) for 48 h.

### 2.3. Experimental Design for Demethylation of Lignin

The DL products were prepared by nucleophilic substitution reaction under atmospheric pressure. Na_2_SO_3_ was used as the demethylation catalyst, and the concentration (weight ratio of NaOH to the weight of lignin) of NaOH in the reaction mixture was 10 wt %. The quantitative evaluation and statistical analysis of the effects of demethylation reaction parameters on the reactivity of DL products were studied through orthogonal experiments, and the orthogonal array L9 (3^4^) was used. Three relevant variables: reaction temperature (t), reaction time (T), and demethylation catalyst dosage (Cd, weight ratio of demethylation catalyst to the weight of lignin) were investigated. During the demethylation process, lignin, demethylation catalyst, NaOH, and distilled water were mixed in a 100 mL flask. The mixture was heated at a given temperature for a given time, stirring in 1000 r/min, and then the mixture was cooled to 40 °C to yield DL products. It was applied to the orthogonal experiment for analysis of the effects of demethylation reaction parameters on relative absorbance of methoxyl groups (RA_Me_), bonding strength, and formaldehyde emission of plywood bonded with the synthesized lignin-based phenolic resins. The investigated factors and the corresponding levels are listed in Table 1.

### 2.4. Structural Characterization of Lignin

Lignin samples were freeze dried at −60 °C for 48 h. Then the freeze-dried samples were milled to 200 mesh meal, and the structure characterization of lignin was performed by FT-IR and ^1^H-NMR spectrophotometers. In a typical test of FT-IR analysis, lignin samples were combined with KBr powder and pressed into pellets. FT-IR spectra of the samples were obtained using a Nicolet 6700 spectrometer (Nicolet Instrument Corporation, Madison, WI, USA) at a resolution of 4 cm^−1^. Each spectrum was recorded in the frequency range of 500–4000 cm^−1^ with 32 scans. The FT-IR spectra were used to calculate relative absorbance of the interested functional groups according to our previous work [12]. Relative absorbance is the ratio between the intensity of the functional group bands. The methoxyl groups appeared at 1461–1463 cm^−1^, and the aromatic ring bands (Ar) presented at 1498–1500 cm^−1^. The bands of aromatic groups were used for normalization and their intensity was set to 1.00, and the relative absorbance of functional groups was calculated according to the following formula:

(1) $$RI\;=\;\frac{I}{I_{\mathrm{Ar}}},$$

where *RI* is the relative absorbance intensity of a specific functional group; *I* is the absorbance intensity of the band that refers to the functional group; and *I*_Ar_ is the absorbance intensity of the aromatic ring band.

The first step in the ^1^H-NMR measurement was the acetylation of lignin aimed to enhance its solubility in organic solvent. Briefly, 2 g of lignin was added to 30 mL of solution mixture of pyridine and acetic anhydride (1:1, *v*/*v*) in a 100-mL conical flask. After stirring at ambient temperature for 48 h, the mixture was treated with 10 times volume of 1 wt % HCl at 0 °C. Finally, the resulting precipitate was filtered, washed with distilled water to neutral pH, and dried at ambient temperature. In the ^1^H-NMR analysis, 0.1 g of acetylated lignin samples and 0.01 g of p-nitrobenzaldehyde, which was used as internal standard, were dissolved in CDCl_3_. The ^1^H-NMR measurement was performed at ambient temperature using a JEOL ECS-400 NMR spectrometer (Tokyo, Japan) at a frequency of 300 MHz with an acquisition time of 3.0 s.

The relative content of specific functional groups was further calculated by ^1^H-NMR spectra based on previous work [26]. The relative content of functional groups was calculated according to the following formula:

(2) $$F\;=\;\frac{\frac{M_{i}}{I_{i}}\times A}{m}\;\times \;100\%,$$

where *F* is the relative content of specific functional groups; *A* is the area of the peak that refers to the functional group; *M*_i_ is weight of internal standard (g); *I*_i_ is the area of the peak that refers to internal standard; and *M* is the weight of lignin (g).

### 2.5. Preparation of Phenolic Resins

The phenol-formaldehyde (PF) resins were synthesized by batch copolymerization using phenol and formaldehyde with a molar ratio of 1:2.2. Phenol, one third of the formaldehyde, and NaOH solution (50 wt %) were mixed in a 100-mL flask in the first stage, and the mixture was heated for 50 min at 85 °C. In the second stage, another one third of the formaldehyde and NaOH solution was added to the flask for 60 min at 85 °C. In the third stage, the rest of the formaldehyde and NaOH solution was added to the same flask for 50 min at 85 °C. In the fourth stage, a small amount of urea and NaOH solution was added to the flask, which was then heated for 30 min at 85 °C. When the viscosity reaches to 100–150 mPa s, the reaction mixture was rapidly cooled to 40 °C to yield the PF resins.

The lignin-phenol-formaldehyde (LPF) resins and the demethylated lignin-phenol-formaldehyde (DLPF) resins were synthesized by batch polymerization according to the molar ratio phenol together with lignin to formaldehyde was 1:2.2 [16]. Half phenol was substituted by lignin according to our previous research [12]. In the first step, phenol, lignin, one-third formaldehyde and NaOH solution (50 wt %) was added to the flask and heated for 50 min at 85 °C. The rest fabrication processes were the same as those for the fabrication of PF resins.

### 2.6. Characterization of Phenolic Resins

The liquid-state ^13^C-NMR spectra of the freeze-dried phenolic resins were recorded on a JEOL ECS-400 NMR spectrometer at a frequency of 75.51 MHz. DMSO-_D6_ was used as the solvent. All of the spectra were recorded at room temperature with a relaxation delay of 8 s over 800 scans. The chemical shifts of each spectrum were accurate to 0.1 ppm and all the samples were directly used for ^13^C-NMR measurement.

The gel time of the phenolic resins was determined in accordance with Chinese National Standard (GB/T 14074.3-2006). Thermogravimetric measurement of the freeze-dried phenolic resins was performed with a Q50 thermogravimetric analyzer (TGA, TA Instruments, New Castle, DE, USA) in a nitrogen atmosphere (flow rate = 60 mL/min) and at a temperature range from room temperature to 600 °C, with a heating rate of 10 °C/min.

### 2.7. Preparation and Characterization of Plywood

Three-layer plywood (400 mm × 400 mm × 4.5 mm) was prepared with single eucalyptus veneers. The veneer was coated with 125–150 g/m^2^ resins each side. First, the glued plywood samples were cold-pressed under 0.8 MPa for 0.5 h, and then hot-pressed at 130 °C under 1.2 MPa for 5 min. Finally, the obtained plywood were stored at ambient conditions for 24 h prior to shear strength testing.

The shear strength was measured as per ASTM D906-98 A. Formaldehyde emissions were measured using the acetylacetone method [15]. In the typical formaldehyde emission test, ten plywood samples with the dimension of 15 cm × 5 cm were placed into a 10-liter glass desiccator contained 300 mL of distilled water at the bottom. After 24 h at 20 °C, the concentration of formaldehyde absorbed in the distilled water was determined by the acetylacetone method with the UV spectrophotometer at a wavelength of 412 nm.

## 3. Results and Discussion

### 3.1. Demethylation and Characterization of Lignin

Demethylation of lignin was conducted under atmospheric pressure and low temperature, aiming to enhance demethylation efficiency and lignin reactivity for the preparation of fast curing bio-based phenolic resins. Na_2_SO_3_ was used as the demethylation catalyst, and three reaction parameters (reaction temperature, reaction time, and demethylation catalyst dosage) were studied. Reference to the experimental design theory, the orthogonal array L9 (3^4^) was selected to arrange the test program (Table 2). Plywood bonding strength is a significant property of phenolic resins, which can be used as an indicator of reaction reactivity between lignin and formaldehyde. In our hypothesis, plywood bonded by LPF resins possess low bonding strength because of the low reactivity of the original lignin, while plywood bonded by DLPF resins would exhibit high bonding strength due to the high reactivity of lignin benefited from the demethylation reaction. Hence, the bonding strength of plywood bonded by phenolic resins was selected as the criterion of each test. 

As seen in Table 2, ten experimental runs were tested, namely experiment 1 to 9, and the optimal combination test. K1, K2, and K3 stand for the estimates value (level 1–3) of bonding strength of the three investigated factors A, B, and C, respectively; k1, k2, and k3 stand for the general average of level 1–3; R stands for the range (the influence of variables on the results). The order of influence of each variable on the bonding strength appears to be A > C > B. Thus, the reaction temperature has the greatest influence and the reaction time has the smallest influence on the demethylation of lignin. The optimum level of each variable is A1, B2, and C3. Therefore, the optimum reaction conditions were as follows: reaction temperature is 80 °C, reaction time is 60 min, and demethylation catalyst dosage is 15%. As seen, the bonding strength of plywood bonded by DLPF resins all meet the standard for exterior-grade panels. In addition, the bonding strength of plywood prepared under the optimum reaction condition (Y1) was the highest, followed by the test 1 (experiment 1 in Table 2). The formaldehyde emission and the relative absorbance of methoxyl groups of each test were also investigated. For most of the tests, the formaldehyde emission of plywood bonded by DLPF resins was below 0.5 mg/L reaching the E_0_ grade, and the DL-Y1 products possessed the lowest relative absorbance values of methoxyl groups. 

In view of the aforementioned discussion, the original lignin, DL-1 (DL products in test 1), and DL-Y1 (DL products in the optimum reaction condition test) were selected for further study. They were characterized by ^1^H-NMR and FT-IR measurement to study its chemical structure changes during the demethylation process. Prior to the ^1^H-NMR analysis, lignin was acetylated to enhance its solubility in organic solvent. The aromatic and aliphatic hydroxyl groups in the nine-carbon unit of lignin were all transformed into acetyl groups with the addition of excess acetic anhydride. Thus, the obtained acetylated lignin could be used for the quantitative analysis of hydrogen-containing functional groups by ^1^H-NMR spectroscopy.

**Figure 1:**
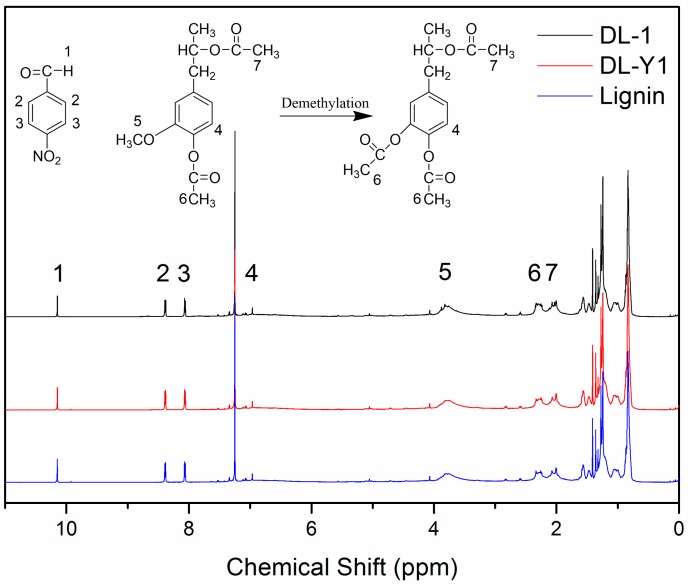
^1^H-NMR (nuclear magnetic resonance) spectra of lignin, DL-1 and DL-Y1.

The ^1^H-NMR spectra of acetylated lignin are shown in Figure 1. The proton signals observed at 10.15 ppm (peak 1) can be attributed to the protons in the –CHO of p-nitrobenzaldehyde, and the proton signals present at 8.39 ppm (peak 2) and 8.07 ppm (peak 3) are related to the aromatic protons in p-nitrobenzaldehyde. Protons attributed to the aliphatic moiety in lignin can be observed between 0.8 ppm and 1.5 ppm; and the protons related to the aromatic protons in S and G units exhibited between 6.0 ppm and 8.0 ppm [27]. It should be pointed out that the proton signals observed at 6.96 ppm (peak 4) were assigned to the active proton at C5 position in the nine-carbon unit of lignin [12]. The existence of such a proton signal (6.69 ppm) indicated that lignin used in this approach possessed active sites for methylolation reaction with formaldehyde. The proton signals at around 3.82 ppm (peak 5) were assigned to the methoxyl groups in lignin, which are closely related to proportion of G: S [27,28]. The protons in –CH_3_ of the aromatic and aliphatic acetyl groups were observed at 2.27 ppm (peak 6) and 2.03 ppm (peak 7), respectively [27]. As can be seen in Figure 1, peak 5 was observed in all ^1^H-NMR spectra, indicating the existence of methoxyl groups in all of the tested lignin. While the decreased intensity of peak 5 in the spectra of DL products indicated that some methoxyl groups were demethylated under the mild reaction condition. Compared to the original lignin, the proton signal intensity of peak 6 slightly increased, which could be attributed to the increase of aromatic acetyl group content in the tested DL products. These phenomena observed in the ^1^H-NMR spectra revealed that with the addition of demethylation catalyst, methoxyl groups in the nine-carbon unit of lignin were attacked by nucleophiles, and some methoxyl groups were converted to phenolic hydroxyl groups by a nucleophilic substitution reaction, resulting in DL products with higher content of phenolic hydroxyl groups and lower content of methoxyl groups. 

A quantitative analysis method was adopted to accurately investigate the chemical structure changes of lignin demethylated under mild condition using Na_2_SO_3_ as the demethylation catalyst. As seen in Figure 1, the intensity and/or the area of peak 2 and peak 3 were similar to each other. Therefore, peak 2 and peak 3 were used as internal standard for the evaluation of specific functional group content. The calculation process and the calculated results for evaluating the relative content of specific functional groups are shown in Table 3. As seen, the aromatic protons in p-nitrobenzaldehyde exhibited similar molar amount in the three tested samples (0.69 m mol/g for lignin, 0.70 m mol/g for DL-1, and 0.71 m mol/g for DL-Y1), which was caused by the same mass fraction of *p*-nitrobenzaldehyde in 1 g of lignin. The methoxyl group content in lignin was 1.93 m mol/g, while the methoxyl group content in DL-1 and DL-Y1 was 1.82 m mol/g and 1.09 m mol/g, respectively. With the demethylation of lignin, the content of aromatic acetyl groups increased from 0.53 m mol/g (acetylated lignin) to 0.58 m mol/g (acetylated DL-1) and 0.76 m mol/g (acetylated DL-Y1), respectively. In addition, the total content of acetyl groups in the acetylated DL was higher than that in the acetylated lignin. As mentioned above, the aromatic acetyl groups emerged by the acetylation reaction of phenolic hydroxyl groups in lignin, and the aliphatic acetyl groups were formed by acetylation reaction of aliphatic hydroxyl groups. Therefore, the content of aromatic and aliphatic acetyl groups in the acetylated lignin was the same with the phenolic and aliphatic hydroxyl group content. Thus, with the addition of demethylation catalyst, the phenolic hydroxyl group content increased from 0.56 m mol/g (lignin) to 0.61 m mol/g (DL-1) and 0.82 m mol/g (DL-Y1), respectively. Furthermore, the total hydroxyl group content in DL was higher than that in lignin. These results confirmed that the methoxyl groups in lignin were transformed in phenolic hydroxyl groups by demethylation reaction under mild condition, and the optimum reaction condition facilitated more rapid demethylation reaction. Wu et al. [24] reported that the methoxyl groups content in soda lignin decreased by about 41.4% after demethylating in autoclave at 220 °C for 10 min. In our approach, the methoxyl groups content decreased by about 43.5%, which was slightly higher than the results reported by Wu et al. In addition, the demethylation of lignin in our approach was performed at mild condition without the use of high pressure and high temperature. Therefore, the demethylation method in this approach had improved demethylation efficiency and the DL products were supposed to have an enhanced reactivity. Moreover, the demethylation of lignin using sodium sulfite as a catalyst under mild condition is an efficient, environmentally friendly, and cost-effect demethylation technique. 

**Figure 2:**
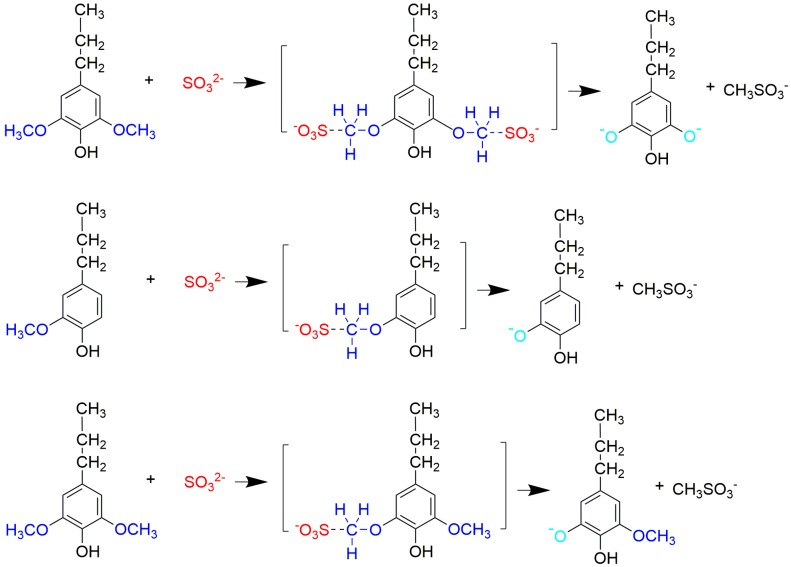
Reaction mechanism of lignin demethylation under mild condition.

Based on the above results, we proposed a demethylation mechanism to illustrate the demethylation of lignin (Figure 2). During the demethylation process, the sulfite ion at the side of methoxyl groups acted as the nucleophilic reagent, and the aryl methyl ethers of lignin compounds were cleaved by nucleophilic substitution reaction, resulting in the removal of methoxyl groups and the formation of phenolic hydroxyl groups. 

**Figure 3:**
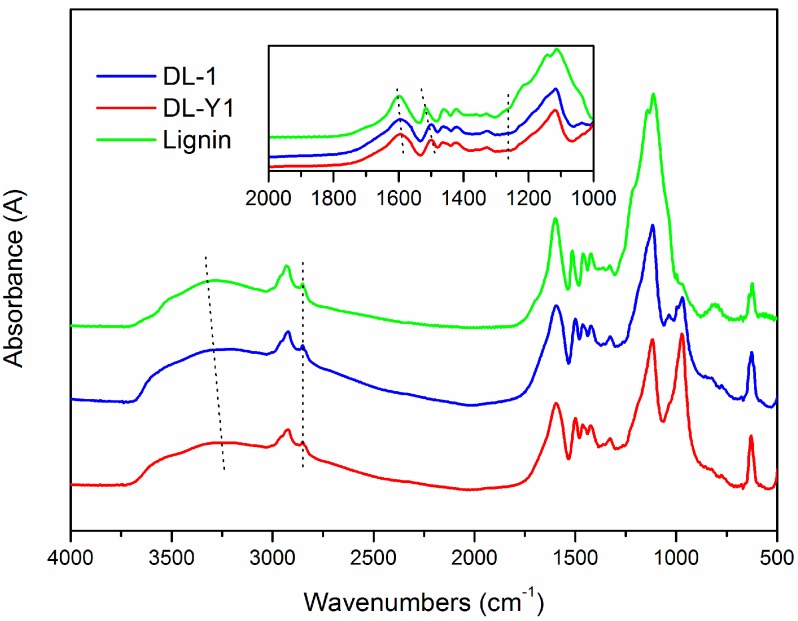
Fourier transform infrared (FT-IR) spectra of lignin, DL-1, and DL-Y1.

To further verify the demethylation mechanism, FT-IR analysis was conducted. The FT-IR spectra (Figure 3) revealed that the main functional groups presented in lignin were similar to that of DL, indicating structural similarity between them. As for the spectrum of lignin, the absorption bands at 3283 cm^−1^ were related to the aromatic and aliphatic –OH groups. The C–H vibration of –CH_2_ and –CH_3_ groups were represented by the band of 2929 cm^−1^, and the bands at 2851 cm^−1^ and 1461 cm^−1^ were assigned to the C–H vibration of –OCH_3_ groups [27]. The presence of absorption bands at 1598 cm^−1^, 1515 cm^−1^ and 1422 cm^−1^ in the spectrum of lignin were assigned to the aromatic ring vibrations of the phenyl-propane skeleton. The peak of 1515 cm^−1^ was attributed to the aromatic skeletal vibrations coupled with C–H plane deformation. While the 1422 cm^−1^ band corresponded to the aromatic ring vibration affected by the nature of the ring substituent and coupled to C–H in plane deformations [12,14]. The aromatic C–H in plane deformation vibration in the syringyl moiety of lignin can be seen at 1329 cm^−1^ and 1113 cm^−1^, while the aromatic C–H out of plane deformation vibration in the guaiacyl moiety of lignin presented at 808 cm^−1^ [27].

As seen in Figure 3, there are some differences between the FT-IR spectra of lignin and DL products. For example, the absorption bands attributed to –OH stretching vibrations that appeared at 3283 cm^−1^ shifted to low wavenumbers (3203–3233 cm^−1^), reflecting the enhanced hydrogen bonding interactions (either –NH and –OH) and the increase of phenolic hydroxyl groups in the lignin polymer networks [29]. In addition, the absorption bands assigned to the aromatic ring vibrations of the phenyl-propane skeleton were shifted from 1598 cm^−1^ and 1515 cm^−1^ to 1595 cm^−1^ and 1499 cm^−1^, respectively. This hypsochromic shift benefited from the cleavage of the –OCH_3_ groups in the nine-carbon unit of lignin, which was consistent with the increase of phenolic hydroxyl groups. The presence of peaks at 1263 cm^−1^ in the spectra of DL was caused by C–O in plane deformation vibration in phenolic hydroxyl groups, which was absent in the spectrum of lignin. Furthermore, the relative absorbance values of –OCH_3_ groups in DL was lower than that in lignin based on a semi-quantitative analysis method, and DL-Y1 possessed the lowest relative absorbance values amongst the tested lignin. The appearance of bands at around 971 cm^−1^ indicated the presence of sulfonic groups in the DL products [30]. The intensity of the peak at 971 cm^−1^ in the spectrum of DL-Y1 was higher than that of DL-1, indicating there were more sulfonic groups in DL-Y1 than that in DL-1. These results confirmed that –OCH_3_ groups were cleaved and the phenolic hydroxyl groups emerged during the lignin demethylaton process under mild reaction condition, which was consistent with the ^1^H-NMR analysis. 

It has been reported that the methoxyl groups at the ortho positions to the aromatic hydroxyl in the nine-carbon unit of lignin will hinder the reactivity of its free phenolic hydroxyl groups. If the –OCH_3_ groups could be removed and demethylated, the demethylated lignin products would have far more free phenolic groups and its reactivity could be significantly enhanced [13]. Previous works reported that methoxyl groups in lignin were removed through a nucleophilic substitution reaction when the aryl methyl ethers were attacked by nucleophiles such as S and HS^−^, resulting in demethylated lignin with lower content of methoxyl groups and higher content of phenolic hydroxyl groups [13,19]. However, the rigid reaction condition obstructed their industrial applications. In this approach, Na_2_SO_3_ was used as the demethylation catalyst, and alkali lignin was demethylated under mild reaction condition. FT-IR and ^1^H-NMR analysis revealed that methoxyl groups in alkali lignin were successfully transformed into phenolic hydroxyl groups by nucleophilic substitution reaction with high demethylation efficiency. The obtained DL products possessed higher phenolic hydroxyl group content and lower methoxyl group content than the original lignin. In addition, the total hydroxyl group content of DL was also higher than that of lignin. It was believed that phenolic hydroxyl groups could activate the free ring positions in lignin, promoting the polymerization of phenolic resins. Therefore, the obtained DL-1, DL-Y1, and lignin were used for the fabrication of fast curing bio-based phenolic resins, and PF resins synthesized by phenol and formaldehyde were used as a control. 

### 3.2. Chemical Structure Characterization of DLPF Resins

**Figure 4:**
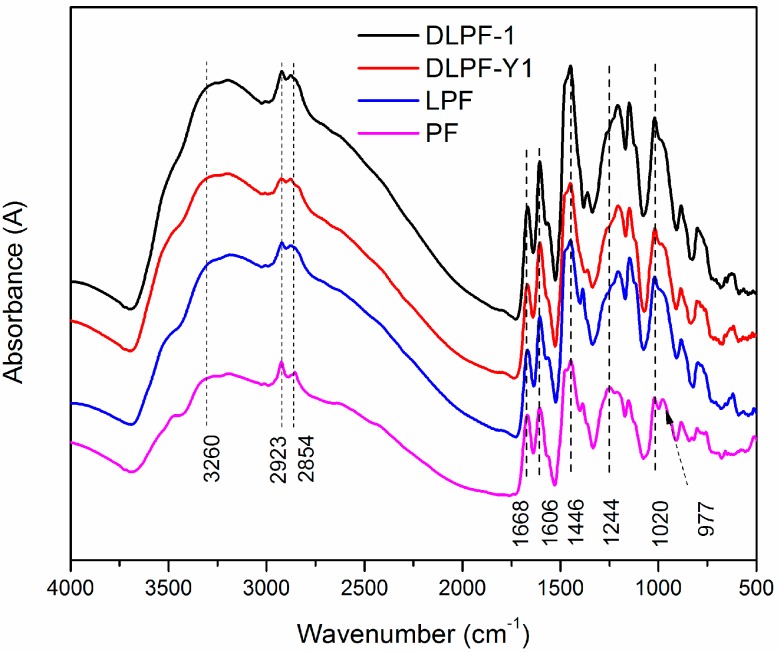
FT-IR spectra of phenol-formaldehyde (PF), lignin-phenol-formaldehyde (LPF), and demethylated lignin-phenol-formaldehyde (DLPF) resins.

To study the structural changes between PF, LPF, and DLPF resins, FT-IR spectra were recorded. As seen in the FT-IR spectra (Figure 4), the functional groups presented in all the phenolic resins were similar to each other, indicating they share a common molecular structure. 

The wide absorption bands at around 3260 cm^−1^ corresponded to –OH bridging groups in all phenolic resins, and the small peaks at the range of 2850–2923 cm^−1^ were associated with the methylene bridges [31]. The absorption bands at 1606 and 1446 cm^−1^ were related to the aromatic ring vibrations of the phenyl-propane skeleton, and bands at 1668 cm^−1^ can be attributed to unconjugated carbonyl groups [15]. The band at 1244 cm^−1^ were ascribed to the presence of C–O stretching vibration of the phenolic hydroxyl groups, while the C-O stretching vibration of aliphatic C–O(Ar), aliphatic C–OH and methylol C–OH were represented by the band of 1020 cm^−1^ [32]. It was observed that the absorption band at 1250 cm^−1^ in the spectra of the LPF and DLPF resins was weaker than that of the PF resins, revealing that there were more C–O stretching vibrations of the phenolic hydroxyl groups in the PF resin than in the LPF and DLPF resins. This was because in the bio-based phenolic resins phenol was partly substituted by lignin containing both a phenolic units and non-phenolic units, resulting in lower phenolic hydroxyl group content [16]. It could be seen that the band at 1020 cm^−1^ of LPF and DLPF resins was broader than that in the PF resins, because three were more methylol-OH and aliphatic-OH in LPF and DLPF resins. This phenomenon was in agreement with previous reports [15,16]. The peak at 977 cm^−1^ was associated with C-H stretching vibration of vinyl in PF resin, which was absent in the spectra of LPF and DLPF resins.

**Figure 5:**
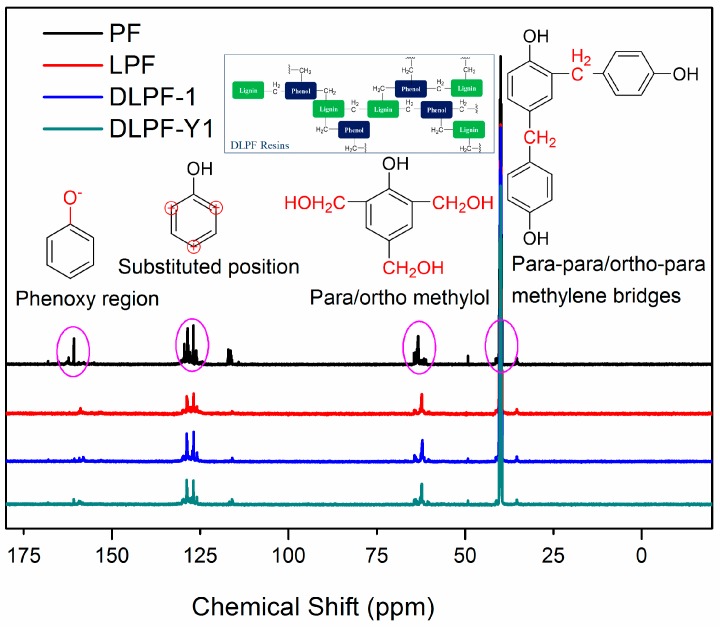
Liquid-state ^13^C NMR spectra of PF, LPF, and DLPF resins.

To identify the effect of different lignin on the functional groups of phenolic resins, liquid-state ^13^C-NMR was used to investigate the difference of chemical shifts between the pure PF resins and the bio-based phenolic resins. The liquid-state ^13^C-NMR spectra of various freeze-dried PF, LPF and DLPF resins are shown in Figure 5. As seen, the ^13^C-NMR spectra of PF resins were similar to that of the bio-based phenolic resins, indicating that the bio-based phenolic resins exhibited molecular structure similar to the PF resins, which agreed with the FT-IR analysis. The chemical shift of 154.0–163.0 ppm was related to phenoxy carbons, and the para alkylated groups and ortho alkylated groups were observed at 160.7–162.8 ppm and 154.4–160.2 ppm, respectively [33]. The dominant chemical shifts at around 129.0 ppm were assigned to the substituted ortho and para carbon sites on aromatic ring. The main reactive sites for the methylolation reaction, the unsubstituted para and ortho carbons, presented at 119.1–120.5 ppm and 115.1–117.8 ppm, respectively [16]. The chemical shifts at 61.1–61.5 ppm were assigned to the methylol groups on the ortho position of phenolic hydroxyl groups, and the peaks observed at 63.3–64.8 ppm were associated with the para methylol groups [33]. Theoretically, methylene ether bridges could be formed during the condensation process of phenolic resins. However, the absence of peaks between 69.0 ppm and 74.0 ppm indicated that there were no methylene ether bridges in the synthesized phenolic resins. The peaks ascribed to the para–para and para–ortho methylene bridges were presented at 39.8–41.1 ppm and 34.7–35.4 ppm, respectively. The peaks at around 50 ppm indicated the presence of methanol for all phenolic resins, which were formed during resin synthesis process from the Canizzaro reaction of formaldehyde [15]. 

For all the phenolic resins, the intensity of para–para methylene bridges at 39.9–41.1 ppm was higher than that of para–ortho methylene bridges at 35.7–36.0 ppm. The intensity of chemical shifts at 63.6–64.2 ppm, ascribed to the para methylol groups, was higher than that of peaks at 62.5–63.2 ppm, which were assigned to the methylol groups on the ortho position of phenolic hydroxyl groups. This phenomenon was caused by the higher reactivity of aromatic hydrogen at the para position of the phenolic hydroxyl groups than that at the ortho position. Compared to the spectrum of PF resins, the chemical shifts presented at 119.1–120.5 ppm and 115.1–117.8 ppm, ascribed to the unsubstituted active sites in the aromatic ring decreased in the spectra of LPF and DLPF resins. This was caused by the incorporation of lignin with low content of the active sites in the phenolic resins. In addition, DLPF resins exhibited higher integral intensity of unsubstituted active sites in the aromatic ring than LPF resins, which was caused by the increase of active sites in DL products derived from the demethylation reaction. The fact that the substituted ortho and para carbon sites signals of DLPF resins were larger than that of LPF resins was caused by the enhanced reactivity of DL products. Furthermore, the integral intensity of methylol groups at 63.3–64.8 ppm and 61.1–61.5 ppm in LPF resins were lower than that in DLPF resins. The para–para methylene bridges (39.8–41.1 ppm) and para–ortho methylene bridges (34.7–35.4 ppm) between phenolic units in the spectra of DLPF resins exhibited higher integral intensity than that in the LPF resins. These phenomena can be ascribed to the higher copolymerization degree of the DLPF resins than the LPF resins, which was caused by the higher reactivity of the DL products than the original lignin.

### 3.3. Physical Properties of DLPF Resins

**Figure 6:**
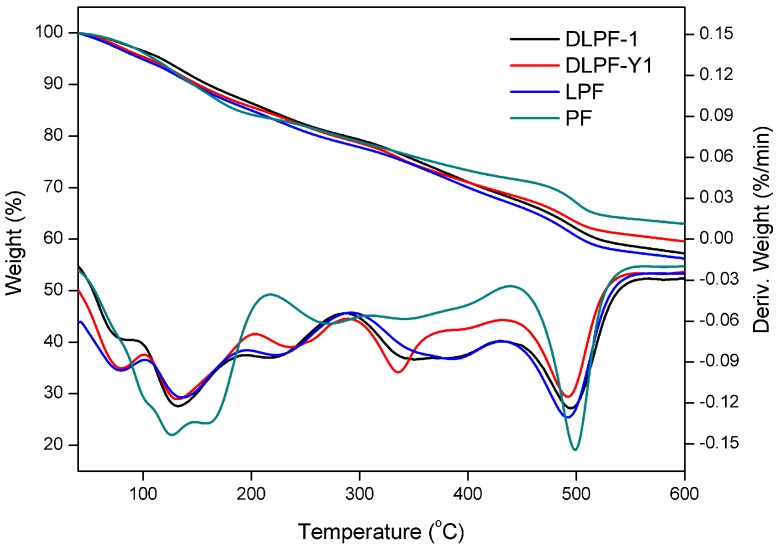
TGA-DTG curves of PF, LPF, and DLPF resins.

To characterize thermal stability of phenolic resins, TGA analysis was carried out under a nitrogen atmosphere. The peak of the derivative thermogravimetry (DTG) curves (T_max_), which can be expressed as the maximum thermal degradation temperature in different thermal events, was used to compare the thermal stability characteristics of phenolic resins [15]. Figure 6 shows the TGA-DTG curves of phenolic resins, and the data of their thermal degradation are given in Table 4.

As seen in the TGA-DTG thermograms of phenolic resins, the degradation can be divided into three steps: post-curing, thermal reforming and ring stripping [16]. The first degradation stage occurred in the range of 110–210 °C. The mass loss in the first stage was attributed to the evaporation of water, which was generated by condensation reaction of the methylol groups [15]. When it comes to the bio-based phenolic resins, the T_max_ of the first degradation stage was in the range of 131–136 °C, higher than the PF resins (124 °C). As seen in this degradation stage, LPF resins and DLPF resins exhibited higher thermal stability than the PF resins. In addition, DLPF resins showed better thermal stability and lower degradation rate than the LPF resins, which was caused by the higher polymerization degree of the DLPF resins.

The second degradation stage is in the range of 210–430 °C. The mass loss in the second stage was due to the loss of water produced from the condensation reaction of phenolic hydroxyl and methylene as well as between two hydroxyl functional groups [16]. It was observed that LPF resins and DLPF resins showed similar thermal stability to the PF resins in the second degradation stage. 

The third degradation stage occurred in the range of 430–570 °C. The mass loss in the third stage arose from the elimination of carbon monoxide and methane formed by the degradation of methylene bridge [12]. The PF resins in the third degradation stage exhibited better thermal stability than the bio-based phenolic resins, because the T_max_ (499 °C) and the weight residue (62.8%) at 600 °C were higher than that of the bio-based phenolic resins. Compared to LPF resins, the DLPF resins had higher T_max_ and greater weight residue at 600 °C, attributing to the higher reactivity of DL. The introduction of DL, which had higher reactivity, facilitating the formation of phenolic resins with higher polymerization degree, thus gave more weight residue at high temperature. 

**Figure 7:**
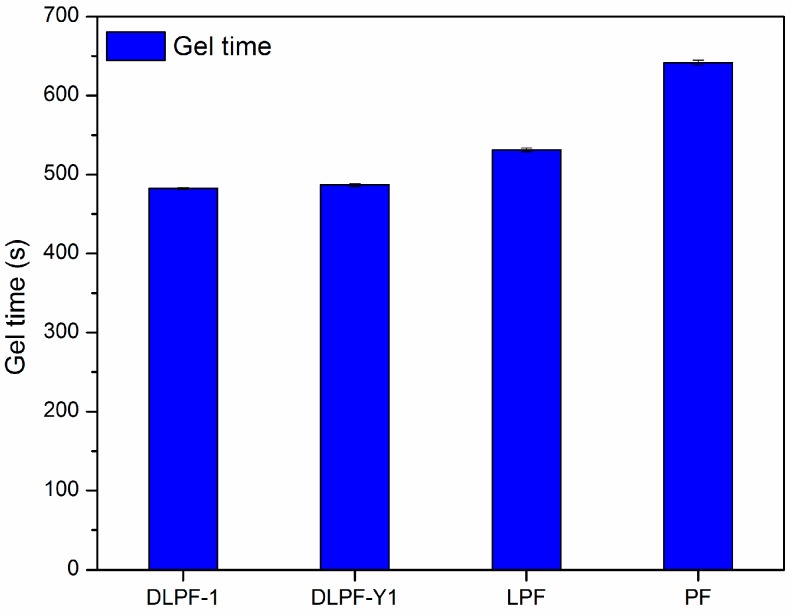
Gel time of PF, LPF, and DLPF resins.

The gel time of various phenolic resins are shown in Figure 7. Three spectrums were tested for each phenolic resin. Gel time was used as an indicator of the curing rate and molecular reactivity of the phenolic resins, because which is generally defined as the time required for phenolic resins to transform from a disorganized liquid into a three-dimensional macromolecular structure under specified conditions [12]. Compared to PF resins, LPF and DLPF resins had shorter gel time and faster curing rate. It is well known that higher molecular weight can result in the shorter gel times [34], hence the faster curing rate of LPF and DLPF resins benefited from the higher molecular weight of lignin over phenol. Among the bio-based phenolic resins, DLPF resins exhibited shorter gel time than the LPF resins. Partial methoxyl groups in the framework of lignin were converted into phenolic hydroxyl groups through demethylation reaction under mild condition, which resulted in increased polymerization degree of the DLPF resins and subsequently their reactivity; hence, DLPF resins had a faster curing rate, as shown in Figure 7.

**Figure 8:**
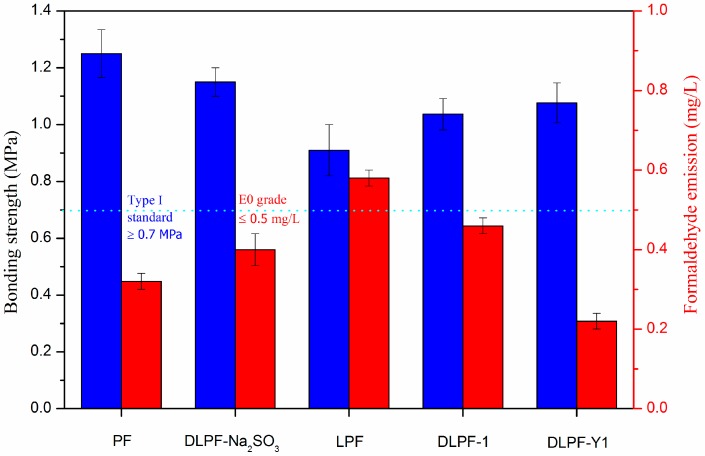
Bonding strength and formaldehyde emission of PF, LPF, and DLPF resins (DLPF-Na_2_SO_3_ was the data reported by Li et al. [12]).

The bonding strength (determined after boiling the plywood samples for 3 h) and the formaldehyde emissions of plywood bonded with the phenolic resins are depicted in Figure 8. Eight spectrums were tested for each kind of plywood for the test of bonding strength, and ten spectrums were tested for each kind of plywood for the test of formaldehyde emission. Plywood bonded with PF resins had higher bonding strength and lower formaldehyde emission than the bio-based LPF resins, because there are many non-phenolic compounds in lignin [34]. In addition, the reactivity of lignin was lower than phenol because phenol has three active sites (two ortho and one para) available for polymerization, while there are two or less active sites on the molecule of lignin. Furthermore, lignin possessed large molecular weight and far greater steric hindrance, which could adversely affect the polycondensation reaction of LPF resins [16]. With the addition of lignin, the bonding strength of plywood decreased from 1.24 MPa to 0.91 MPa, but still meet the standard for exterior-grade plywood panels (0.7 MPa). As seen in Figure 8, plywood bonded with the DLPF resins had higher bonding strength and lower formaldehyde emission than the LPF resins, which was caused by the enhanced reactivity of DL products. In addition, plywood bonded with DLPF-Y1 resins possessed the highest bonding strength and the lowest formaldehyde emission among the bio-based phenolic resins. This phenomenon can be explained by the highest phenolic hydroxyl group content in the DL-Y1 products demethylated under the optimum reaction condition. It was much encouraged that the formaldehyde emission of plywood decreased from 0.58 mg/L to 0.22 mg/L after the demethylation of lignin at the optimum condition. This is an advantage to achieve the purpose of preparing ecofriendly phenolic resins by using lignin to replace petrochemical materials. Compared to plywood bonded with DLPF-Na_2_SO_3_, plywood bonded with DLPF-Y1 had lower bonding strength. It should be pointed out that plywood bonded with DLPF-Na_2_SO_3_ was hot pressed at 130 °C for 7 min, while the plywood bonded with DLPF-Y1 was hot pressed at 130 °C for 5 min. That is why the bonding strength of plywood bonded with DLPF-Y1 was lower than that of plywood bonded with DLPF-Na_2_SO_3_. The formaldehyde emission of plywood bonded with DLPF-Y1 was lower than that of plywood bonded with DLPF-Na_2_SO_3,_ which may be caused by the enhanced reactivity of lignin demethylated at the optimum condition. The increased bonding strength and the decreased formaldehyde emission of plywood bonded with DLPF resins lead us to the conclusion that the DL products had enhanced reactivity, benefited from the removal of methoxyl groups and the formation of phenolic hydroxyl groups during the demethylation process. In addition, the improved properties of DLPF resins indicated that the demethylation technique in this approach had an enhanced demethylation efficiency, which could be confirmed by the ^1^H-NMR and FT-IR analysis.

## 4. Conclusions

Agricultural crop-based lignin was demethylated under mild reaction condition to enhance the demethylation efficiency and the reactivity of lignin for the preparation of fast curing bio-based phenolic resins. The demethylation reaction mechanism was studied by the characterization of lignin and DL products through FT-IR and ^1^H-NMR spectra, and the demethylation condition was optimized according to the reactivity of the DL products. Our results demonstrated that the demethylation technique in this approach had satisfactory demethylation efficiency. The methoxyl group content of DL-Y1 products decreased from 1.79 m mol/g to 0.94 m mol/g, and the phenolic hydroxyl group content increased from 0.51 m mol/g to 0.71 m mol/g. Some methoxyl groups were converted to phenolic hydroxyl groups by a nucleophilic substitution reaction, resulting in DL products with high reactivity. In addition, the DL products demethylated under the optimum reaction condition possessed the greatest reactivity among the tested lignin samples. 

Compared to LPF resins, DLPF resins exhibited better performances such as faster curing rate, shorter gel time, higher reactivity, and lower formaldehyde emission. In addition, DLPF-Y1 resins exhibited the best performances among the synthesized bio-based phenolic resins.

## Figures and Tables

**Table 1:** Investigated factors and the corresponding levels.

Factors	Parameters	Levels		
		1	2	3
**A**	Temperature (°C)	80	90	100
**B**	Time (min)	30	60	90
**C**	Catalyst dosage (%)	5	10	15

**Table 2:** Bonding strength and formaldehyde emission of phenolic resins, and relative absorbance values of functional group (*RI*_Me_) of lignin.

Experiment ID	A	B	C	Bonding strength (MPa)	Formaldehyde emission (mg/L)	*RI*_Me_
1	1	1	1	1.01 ± 0.08	0.48 ± 0.01	0.941
2	1	2	2	0.95 ± 0.10	0.13 ± 0.05	0.947
3	1	3	3	0.83 ± 0.06	0.28 ± 0.02	0.944
4	2	1	3	0.89 ± 0.11	0.38 ± 0.04	0.961
5	2	2	1	0.77 ± 0.20	0.15 ± 0.04	0.965
6	2	3	2	0.92 ± 0.07	0.09 ± 0.02	0.962
7	3	1	2	0.62 ± 0.14	0.66 ± 0.03	0.960
8	3	2	3	0.93 ± 0.08	0.79 ± 0.03	0.980
9	3	3	1	0.82 ± 0.11	0.37 ± 0.05	0.936
K1	2.79	2.52	2.60	-	-	-
K2	2.58	2.65	2.49	-	-	-
K3	2.37	2.57	2.65	-	-	-
k1	0.93	0.84	0.87	-	-	-
k2	0.86	0.88	0.83	-	-	-
k3	0.79	0.86	0.88	-	-	-
R	0.14	0.04	0.05	-	-	-
Order	A > C > B			-	-	-
Optimal level	A1	B2	C3	-	-	-
Optimal combination	A1B2C3			1.07 ± 0.12	0.22 ± 0.03	0.927

**Table 3:** The ^1^H-NMR analysis of acetylated lignin.

Sample ID	Chemical shift (ppm)	8.39	8.07	6.96	3.82	2.27	2.03
	Proton arrangement	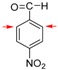	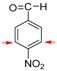	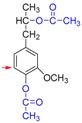	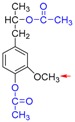	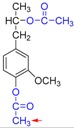	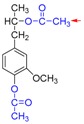
DL-1	Integral area of chemical shifts	1.23	1.29	0.74	4.91	1.65	2.21
	Integral area of one proton in each functional group	0.63		0.74	1.64	0.55	0.74
	Relative integral area of proton	1.00		1.17	2.60	0.87	1.17
	Molar amount of functional groups in 1 g acetylated lignin (m mol/g)	0.66		0.78	1.72	0.58	0.78
	Molar amount of functional groups in 1 g lignin (m mol/g)	0.70		0.82	1.82	0.61	0.82
DL-Y1	Integral area of chemical shifts	1.68	1.72	0.78	3.89	2.93	3.68
	Integral area of one proton in each functional group	0.85		0.78	1.3	0.98	1.23
	Relative integral area of proton	1.00		0.92	1.53	1.15	1.45
	Molar amount of functional groups in 1 g acetylated lignin (m mol/g)	0.66		0.61	1.01	0.76	0.96
	Molar amount of functional groups in 1 g lignin (m mol/g)	0.71		0.65	1.09	0.82	1.03
Lignin	Integral area of chemical shifts	1.52	1.64	0.73	9.65	1.92	1.28
	Integral area of one proton in each functional group	0.79		0.73	2.22	0.64	0.43
	Relative integral area of proton	1.00		0.92	2.81	0.81	0.54
	Molar amount of functional groups in 1 g acetylated lignin (m mol/g)	0.66		0.61	1.85	0.53	0.36
	Molar amount of functional groups in 1 g lignin (m mol/g)	0.69		0.63	1.93	0.56	0.37

Note: Assume that the total mass of acetylated lignin is 1.0 g, then the acetylated lignin containing lignin; M_DL-1_ = 1 − (0.58 + 0.78) × 10 − 3 × 42 = 0.943; M_DL-Y1_ = 1 − (0.76 + 0.96) × 10 − 3 × 42 = 0.928; M_Lignin_ = 1 − (0.53 + 0.36) × 10 − 3 × 42 = 0.962.

**Table 4:** Thermal properties of phenolic resins.

Sample ID	First thermal event T_max_ (°C)	Second thermal event T_max_ (°C)	Third thermal event T_max_ (°C)	Weight residue (%)
PF	124	343	499	62.8
LPF	136	385	492	55.9
DLPF-1	132	350	494	57.2
DLPF-Y1	131	336	492	59.4
